# ‘Deconstructing Stereotypes to Build Consent’: Evaluation of a Project on Social and Sexual Relationships in Adolescence

**DOI:** 10.3390/bs15091275

**Published:** 2025-09-18

**Authors:** Elisa Berlin, Angela Fedi, Elena Ciampi, Caterina Di Chio, Mélodie Husquin, Ivan Luppino, Mara Martini, Chiara Rollero

**Affiliations:** 1 Department of Psychology, University of Turin, 10124 Turin, Italy; angela.fedi@unito.it (A.F.); melodie.husquin@gmail.com (M.H.); mara.martini@unito.it (M.M.); chiara.rollero@unito.it (C.R.); 2 AMAE, 10121 Turin, Italy; ele.ciampi@gmail.com (E.C.); caterina.dichio@gmail.com (C.D.C.); ivan.luppino@gmail.com (I.L.)

**Keywords:** teen dating violence, sexism, sexual double standard, body surveillance, school intervention

## Abstract

Evidence from the international literature indicates alarming prevalence rates associated with various forms of intimate partner violence since adolescence. To prevent gender-based violence and increase psychological well-being in intimate relationships, both the scientific literature and policy makers agree on the importance of implementing specific prevention and education programs targeting adolescents. The purpose of this longitudinal study was to evaluate the impact of an educational intervention to promote awareness of one’s relationship with one’s own body, stereotypes related to gender and sexuality, and the issue of sexual consent. Participants were adolescents aged 15–16 years who filled a questionnaire prior to the start of the intervention (Time 0, N = 192, 55.7% male) and two weeks following its conclusion (Time 1, N = 178, 53.9% male). Results indicate that compared to Time 0, after participation, body surveillance, benevolent sexism, and endorsement of the sexual double standard decreased, while no significant effect emerged in relation to the issue of sexual consent. Implications for research and intervention are discussed, with the goal of providing useful guidance for those implementing interventions for young people to address intimate partner violence and promote relationship well-being.

## 1. Introduction

The onset of individuals’ dating relationships commonly takes place during adolescence, a developmental stage that plays a crucial role in shaping sexual attitudes, identity, and psychological adjustment (Collins, 2003; Javidi et al., 2020). However, adolescence is also recognized as a period when the first instances of intimate partner violence (IPV) may arise (Lozano-Martínez et al., 2022; Vagi et al., 2015), potentially resulting in serious short- and long-term harm to victims’ well-being. In this framework, the concept of teen dating violence (TDV) refers to psychological, physical, and sexual abuse occurring within romantic relationships among adolescents aged 13 to 19 (Centers for Disease Control and Prevention, 2020). Such abusive behaviors can occur in person as well as through digital means, including persistent messaging or the non-consensual distribution of sexually explicit content (Centers for Disease Control and Prevention, 2020; Cutter-Wilson & Richmond, 2011).

International studies investigating TDV report concerning prevalence rates. In Spain, nearly 30% of students admitted to perpetrating at least one form of dating abuse, and 35% reported being victims (Lozano-Martínez et al., 2022). Similarly, over a third of Canadian teens experienced psychological TDV in a year (Hébert et al., 2017), while 20% of U.S. adolescents faced electronic dating abuse (Hinduja & Patchin, 2021). In addition, around 20% of girls aged 16–17 who have been in a relationship reported having experienced psychological abuse, such as insults, blaming, and intimidation (Díaz-Aguado Jalón, 2022). Consistent with international data on gender-based violence (European Commission, 2022), girls are disproportionately affected by physical and sexual victimization, while boys more frequently engage in controlling and coercive behaviors (Clayton et al., 2023; Malhi et al., 2020; Wincentak et al., 2017).

Scholars have consistently documented a significant relationship between IPV and long-term physical and mental health issues, including anxiety, depression (Johnson et al., 2014), and eating disorders (Ackard et al., 2007). Girl victims are also more likely to engage in risky behaviors like unprotected sex (Teitelman et al., 2008), binge drinking (Foshee et al., 2013), and substance abuse (Ackard et al., 2007; Parker & Bradshaw, 2015). TDV has also been associated with self-harm and suicidal tendencies (Holmes & Sher, 2013; Quarshie, 2021). In school settings, female victims often report low self-esteem, concentration difficulties, and social withdrawal (Díaz-Aguado Jalón et al., 2014). Lastly, TDV increases the risk of experiencing abusive relationships later in life (Exner-Cortens et al., 2017; Teitelman et al., 2008).

### 1.1. Psychosocial Factors Associated with TDV

Several psychosocial factors have been identified as influential in shaping the perception of gender-based abuse, including self-objectification processes, sexism, and sexual double standards (SDSs). Self-objectification refers to a form of self-perception in which individuals prioritize their body’s appearance over its function, thereby conceptualizing themselves as a collection of body parts rather than as a whole (Daniels et al., 2020; Fredrickson & Roberts, 1997). This phenomenon frequently manifests as persistent body surveillance, defined as the continual monitoring of how closely one’s appearance conforms to culturally valued ideals (Fredrickson & Roberts, 1997; Rollero, 2022). In recent decades, the rising prevalence of social media use has been implicated in the increase in an objectifying self-perspective among both women and men (Ozimek et al., 2023). However, body surveillance is particularly widespread among young women and adolescent girls, with significant repercussions for their physical, psychological, and social well-being (Skowronski et al., 2021; Tiggemann & Slater, 2015). In particular, body surveillance appears to be positively correlated with heightened appearance anxiety, and a higher risk of developing eating disorders, depression, and sexual dysfunction (Moradi & Huang, 2008; Saunders & Eaton, 2018; Wollast et al., 2019). Furthermore, research has identified a direct link between IPV victimization and body surveillance. In this regard, drawing on the self-objectification framework, Davidson and Gervais (2015) examined the relations between IPV and body image-related variables, highlighting that having experienced IPV directly predicted individual levels of body surveillance. In addition, self-objectification significantly mediated the relationship between IPV and body surveillance, while body surveillance significantly mediated the relationship between IPV and body shame (i.e., a form of dissatisfaction toward one’s own body). Overall, women with a history of IPV appear to be more likely to experience body surveillance and body shame compared to those without such experiences (Davidson & Gervais, 2015).

Moreover, the relationship between sexism and individuals’ attitudes toward IPV has been widely investigated drawing on ambivalent sexism theory (Glick & Fiske, 1996). According to this framework, on the one hand, the display of overtly negative attitudes toward women (i.e., hostile sexism) can lead to minimizing the severity of gender-based abuse, including the damaging consequences suffered by victims; on the other hand, viewing women as pure and in need of protection (i.e., benevolent sexism) may foster victim-blaming attitudes when traditional gender norms are perceived as violated (Angelone et al., 2021; Chapleau et al., 2007; Rollero & Tartaglia, 2019). A direct association has been found between the aforementioned dimensions and adolescents’ perception of TDV. Specifically, hostile sexism is linked to a greater likelihood to perpetrate TDV (Fernández-Fuertes et al., 2018; Madrona-Bonastre et al., 2023), while benevolent sexism supports the justification of such violence (Fasanelli et al., 2020).

Furthermore, SDS refers to the tendency to evaluate identical sexual behaviors differently, depending on whether they are exhibited by males or females (M. D. C. Gómez Berrocal et al., 2019; C. Gómez Berrocal et al., 2022). Among adolescents, the belief that girls and boys should adhere to distinct sexual norms—emphasizing modesty for girls and sexual assertiveness for boys—has notable implications for individuals’ sexual behavior and psychosocial well-being. For example, early adolescent girls who reported having engaged in sexual intercourse experienced a significant decline in peer acceptance over time, whereas their male counterparts saw a significant increase (Kreager et al., 2016). Consistently, having a higher number of sexual partners is positively associated with peer acceptance among boys, but negatively associated with peer acceptance among girls (Kreager & Staff, 2009). The display of SDS positively correlates with the endorsement of ambivalent sexist beliefs (Zaikman & Marks, 2014). Moreover, it significantly influences both the perception and the propensity to perpetrate gender-based violence, particularly in the context of adolescent dating relationships (Guerra-Marmolejo et al., 2021; Martínez-Gómez et al., 2021).

In addition, adolescents’ attitudes toward TDV are closely intertwined with their understanding of sexual consent, defined as “one’s freely given verbal or nonverbal communication of their sober and conscious feelings of willingness to engage in a particular sexual behavior, with a particular person, within a particular context” (Willis et al., 2019, p. 227). Indeed, sexual consent represents the determining factor between “just” sexual intercourse and sexual abuse, thereby playing a crucial role in the development of safe and respectful sexual relationships (Fenner, 2017). The ability to communicate internal consent (i.e., the subjective experience of willingness to engage in sexual activity; Jozkowski et al., 2014) has also been linked with enhanced sexual satisfaction and enjoyment, especially among young women (Marcantonio et al., 2020). However, although adolescents—particularly girls—appear to endorse supportive attitudes toward affirmative consent (Javidi et al., 2020), they often exhibit limited sexual negotiation skills, which remain heavily influenced by traditional gender norms (Mfeka-Nkabinde et al., 2024). In particular, notable gender discordance persists in how sexual consent and refusal are both communicated and interpreted, particularly concerning the reliance on verbal vs. non-verbal cues (Richards et al., 2022; Righi et al., 2021).

Holding positive attitudes toward mutual sexual consent has been associated with increased sexual well-being and overall psychological adjustment (Espinosa-Hernández et al., 2024). In addition, school-based interventions that address sexual communication and challenge gender stereotypes have been identified as protective factors, reducing the risk of both perpetration and victimization in various forms of gender-based violence (DeGue et al., 2014) and increasing adolescents’ willingness to intervene in situations involving sexual aggression (Shekar et al., 2020). In this regard, comprehensive sexuality education programs that incorporate discussions on consent, respect, and emotional literacy may be crucial in fostering the development of safe and respectful relationships among adolescents (e.g., Foshee et al., 2005). However, the implementation of such programs is rarely accompanied by scientific evaluation, thereby limiting the ability to demonstrate their effectiveness and hindering their integration into established public health strategies.

### 1.2. The Use of Psychodrama in Educational Settings

Classical psychodrama (action of the soul, from psyche, and drama) is an experiential, action-based approach conceptualized by psychiatrist Jacob Levi Moreno (Lim et al., 2021). Psychodrama promotes symmetrical participation and dialogic interaction within the group, aligning with socio-constructivist principles of learning (Watson, 2001). Within this framework, the psychodramatist assumes the role of facilitator, fostering a learning environment oriented toward spontaneity, mutual recognition, and co-construction of meaning. Participants are thus encouraged to articulate their “naïve theories,” personal beliefs, and emotional responses in a protected, non-judgmental space that supports both expression and active listening. Through guided reflection and reformulation by the facilitator, individual contributions are integrated into a shared body of knowledge, constructed collaboratively within the group.

Psychodrama and/or other experiential methods have been increasingly applied in school-based interventions aimed at promoting socio-emotional competencies and preventing aggressive behaviors among students. Previous studies have shown that psychodrama can reduce school-related anxiety and disruptive behaviors among adolescents, while fostering empathy, self-awareness, and mental resilience (Giannakopoulos et al., 2012; Ulusoy et al., 2023). Moreover, school-based experiential programs using active, participatory approaches appear to be particularly effective in improving social and emotional skills, while reducing aggressive and/or violent behaviors (Durlak et al., 2011; Healy et al., 2020). Within the field of TDV prevention, multi-level interventions that engage schools, families, and communities have been identified as the most promising strategies to promote change (De Koker et al., 2014). Building on this evidence, our intervention integrates psychodrama-based techniques to stimulate critical reflection on TDV-related attitudes and myths, thereby fostering awareness, encouraging constructive dialogue among peers, and promoting the internalization of healthier beliefs and behaviors.

### 1.3. The Project

The project “Growing Up as Equals: Deconstructing Stereotypes to Build a New Consent” builds on a previous initiative titled “Getting Excited, Falling in Love, Experiencing Pleasure: A Journey Towards Awareness.” Both projects were designed for third-year upper secondary school students according to the Italian education system. “Growing Up as Equals” was developed and implemented by the association AMAE APS in collaboration with various professionals, including a midwife sexual health consultant. “Amae” is a Japanese term that refers to the emotional state of comfort and well-being experienced when someone acknowledges their need for support and entrusts themselves to the care of another. The project aims to promote awareness and prevent gender-based violence, with a particular focus on romantic relationships during adolescence.

The Psychology Department of the University of Turin contributed to the evaluation of the project by designing a specific questionnaire and analyzing the data. The questionnaire was administered prior to the start of the sessions (T0) and two weeks following their conclusion (T1).

Between October 2024 and March 2025, a team of psychologists and trainers from AMAE conducted the intervention across eleven third-year classes: eight from Scientific High Schools (both Traditional and Applied Sciences tracks) and three from Technical Institutes. The intervention comprised four sessions of two hours each per class. Three sessions were facilitated by professionals with expertise in psychology, psychotherapy, and/or psychodrama, while one session (the third one) was led by a midwife with additional qualifications as a sexual consultant.

Overall, the purpose of the “Growing as Equals” project was to create a relational space for dialogue and exchange, where each person could feel listened to and acknowledged, experiencing a sense of active participation in the process. Active and participatory methods were employed (e.g., interactive discussions, stories and narratives to identify with, role-playing and role training, photographic storytelling techniques, body awareness). These methods were intended to encourage the emergence of questions and doubts, while also facilitating a temporary state of cognitive disorientation and the subsequent development of new insights and more functional ways of relating to oneself and others (Menzies et al., 2022).

In the first session, for example, four cardboards were placed in the corners of the classroom. Each one displayed a type of violence: physical, psychological, economic, and sexual. In the center of the room, an additional cardboard was placed, indicating witnessed (or secondary) violence. The facilitator read various statements aloud to the class, each describing a situation about violence, and asked the participants to physically position themselves near the cardboard that corresponds to the type of violence portrayed. *[Students frequently recognized that multiple forms of violence, such as physical and psychological, can coexist in the same situation. The concept of witnessed violence, though less familiar, was often reported as being particularly impactful and emotionally significant].*

In the second session, the group engaged in an in-depth exploration of the sexual objectification of bodies. Through the analysis of various advertisements, participants were encouraged to identify those that depict the body, particularly its features and parts, in a reductive and objectifying manner, primarily for commercial purposes. This exercise led to a critical reconstruction of dominant representations of male and female bodies, as well as a reflection on the impact of objectification on self-image and on the internalization of unrealistic body ideals, often associated with anxiety and frustration.

The third session, led by a midwife and sexual health consultant, had the goal of sharing knowledge about the anatomy and physiology of the genital organs, along with a sexuality map, to broaden perspectives on the sexual experience. The discussion also addresses topics such as the negotiation of choices like contraception and the concept of consent in sexual activity.

In the fourth and final session, which focused on the topic of consent, short stories were used as a starting point for discussion. Working in small groups, students read these stories and were invited to identify the characters’ physical sensations, emotions, and thoughts, as well as to reflect on the question “In this story, was consent respected?”. The stories, drawn from real-life situations experienced by adolescents, addressed scenarios linked to the so-called “rape pyramid,” including street harassment and sexually inappropriate remarks.

Beyond the specific objectives of each session, all activities were designed to contribute to the broader aim of deconstructing gender stereotypes (Phillips & De Roos, 2025; Santoniccolo et al., 2023) and promoting open, flexible models of identity and relationships. These models support the expression of each individual’s uniqueness and encourage forms of interaction grounded in mutuality and respect. This process is essential for fostering healthy and mutually respectful interpersonal and sexual relationships.

### 1.4. The Current Study: Objectives and Hypotheses

To assess the impact of the educational intervention described above, a longitudinal study was conducted. Drawing on the previously outlined literature, the study aimed to examine four primary dimensions.

First, we sought to investigate whether engagement in the intervention influenced individual levels of body surveillance. Therefore, we compared participants’ body surveillance before and after the program. We hypothesized the following:

**H1.**  *Participation in the project would reduce individual levels of body surveillance.*

Our second goal was to evaluate the effect of participation in the intervention on participants’ sexist attitudes. Specifically, we assessed individual levels of both hostile and benevolent sexism before and after engagement with the program. We hypothesized the following:

**H2.**  *Participation in the project would lead to a reduction in both hostile (H2a) and benevolent (H2b) sexism.*

The third aim was to examine the intervention’s effect on individual levels of SDS. To this end, participants’ SDS scores were measured prior to and following their involvement in the project. We expected the following:

**H3.**  *SDS scores would decrease after participation in the intervention.*

Finally, the fourth objective was to explore the intervention’s effect on attitudes toward sexual consent. To address this aim, we assessed participants’ attitudes toward establishing sexual consent before and after the intervention. We hypothesized the following:

**H4.**  *Participation in the project would enhance positive attitudes toward establishing sexual consent.*

## 2. Materials and Methods

### 2.1. Participants and Procedure

A formal agreement with the high school involved had been signed. Prior to participating in the project (T0) and two weeks after the final activity (T1), all students from the classes involved in the educational initiative were invited to complete an online survey. The provided link directed them to a secure and anonymous questionnaire platform. Before starting the survey, both students and their parents reviewed an informed consent form and signed a privacy agreement. Questionnaires at T0 and T1 were administered in class during regular school hours. In accordance with the Declaration of Helsinki, participants were informed that their involvement was voluntary and that they could withdraw from the study at any point. No incentives or payments were offered for participation. The Ethics Committee of the University of Turin, Italy approved the study protocol (protocol number 0454951—25 July 2024).

Participants were 192 students (55.7% male, 42.2% female, and 2.1% non-binary/prefer not to say). They all attended the third year of a high school in Turin, Italy. At T1, 178 students responded (53.9% male, 39.9% female, and 6.2% non-binary/prefer not to say). The 14 students who did not take part in the T1 survey were absent from school on that specific day. However, their absence was due to incidental reasons and was not related to an explicit withdrawal or refusal to participate in the study.

### 2.2. Measures

Data were collected by means of a self-report questionnaire, which took approximately 20 min to complete.

In both waves, the questionnaire included the following measures:

1. The Body Surveillance subscale of the Objectified Body Consciousness Scale (McKinley & Hyde, 1996). It measures the extent to which individuals habitually think about how their body looks to others, rather than how it feels or functions. This subscale consists of 8 items (e.g., “During the day, I think about how I look many times”, α at T0 = 0.82, 95% CI [0.78, 0.86], α at T1 = 0.84, 95% CI [0.80, 0.88]) rated on a 7-point scale ranging from 1 (strongly disagree) to 7 (strongly agree). Higher scores indicated that participants were more likely to engage in body surveillance.
2. The short version of the Ambivalent Sexism Inventory (Glick & Fiske, 1996; Rollero et al., 2014) including 12 items measuring benevolent sexism (6 items, e.g., “Women should be cherished and protected by men”, α at T0 = 0.69, 95% CI [0.62, 0.76], α at T1 = 0.75, 95% CI [0.69, 0.81]) and hostile sexism (6 items, e.g., “Once a woman gets a man to commit to her, she usually tries to put him on a tight leash”, α at T0 = 0.85, 95% CI [0.81, 0.89], α at T1 = 0.88, 95% CI [0.84, 0.92]). The items were rated on a 6-point point Likert-type scale ranging from 0 (strongly disagree) to 5 (strongly agree). Higher scores denoted higher levels of sexism.
3. The Acceptance of Sexual Double Standard subscale of the Sexual Double Standard Scale (M. D. C. Gómez Berrocal et al., 2019). It includes 9 items assessing the endorsement of the sexual double standard (e.g., “It’s worse for a woman to sleep around than it is for a man”, α at T0 = 0.75, 95% CI [0.69, 0.81], α at T1 = 0.81, 95% CI [0.76, 0.86]). The items were rated on a 4-point scale ranging from 0 (strongly disagree) to 3 (strongly agree). Higher scores indicated that participants held higher levels of sexual double standards.
4. The Lack of Perceived Behavioral Control and the Positive Attitudes toward Establishing Consent subscales of the Sexual Consent Scale-Revised (Humphreys & Brousseau, 2010; Rollero et al., 2023). Each subscale contains 11 items (Lack of Perceived Behavioral Control, e.g., “I am worried that my partner might think I’m weird or strange if I asked for sexual consent before starting any sexual activity”, α at T0 = 0.89, 95% CI [0.86, 0.92], 95% CI [0.86, 0.92], α at T1 = 0.88, 95% CI [0.85, 0.91]; Positive Attitudes toward Establishing Consent, e.g., “I feel that sexual consent should always be obtained before the start of any sexual activity”, α at T0 = 0.89, 95% CI [0.86, 0.92], α at T1 = 0.91, 95% CI [0.88, 0.94]). The items were rated on a 7-point point Likert-type scale ranging from 1 (strongly disagree) to 7 (strongly agree). Higher scores in the Lack of Perceived Behavioral Control subscale reflected decreased perceived control during sexual activity, while higher scores in the Positive Attitudes toward Establishing Consent subscale indicated major propensity to establish explicit sexual consent.

### 2.3. Data Analyses

Prior to conducting analyses, we assessed the normality of the data using the Shapiro–Wilk test. Results indicated that all variables met the assumption of normality (*p* > 0.05), supporting the use of parametric tests. Then, we carried out zero-order correlations between scales and independent sample *t*-tests to assess gender differences on the study variables at T0. The same analyses were replicated on scores reported at T1. Finally, paired sample *t*-tests were performed to assess whether participants’ scores on the investigated variables changed between T0 and T1. Listwise deletion was applied for analyses where missing data occurred. In addition, non-binary students were excluded from the gender-based analyses due to insufficient sample size for meaningful statistical comparison. However, they were included in all other analyses that did not involve gender as a variable. All statistical analyses were carried out using IBM SPSS Statistics version 28.0 software.

## 3. Results

As shown in Table 1, at Time 0, body surveillance was positively related to favorable attitudes toward consent and negatively related to hostile sexism. In turn, hostile sexism was positively linked to benevolent sexism, sexual double standard, and lack of perceived behavioral control, and negatively linked to positive attitudes toward consent. Lack of perceived behavioral control was associated with sexual double standard, whereas positive attitudes toward establishing consent were negatively related to sexual double standard and lack of perceived control.

Concerning gender differences on the study variables at Time 0, as shown in Table 2, women outscored men on body surveillance and positive attitudes toward establishing consent. On the contrary, men reported higher levels of hostile sexism, sexual double standard, and lack of perceived behavioral control. No significant gender differences emerged in relation to benevolent sexism.

Zero-order correlations between scales were then conducted on scores at Time 1. As shown in Table 3, the correlations between the variables were essentially analogous to those observed at T0. The only discrepancy observed was that at T1; the correlation between body surveillance and positive attitudes toward establishing consent was no longer significant.

As seen in Table 4, gender differences at Time 1 were analogous to those reported at Time 0.

Finally, paired sample *t*-tests were conducted to assess whether participants’ scores on the investigated variables changed between T0 and T1. As can be seen in Table 5, the results showed that body surveillance (H1), benevolent sexism (H2b), and acceptance of sexual double standard (H3) decreased. In contrast, hostile sexism (H2a), lack of perceived behavioral control, and positive attitudes toward establishing consent (H4) did not change substantially.

## 4. Discussion

This study aimed to evaluate the effectiveness of an intervention that focused on the importance of consent in intimate relationships with adolescents aged 15–16 years, attending the third year of upper secondary school. The study analyzed the relationship between multiple dimensions related to TDV and compared participants scores before and after the intervention. Most of our initial hypotheses were confirmed, suggesting the effectiveness of the school-based intervention in reducing dimensions that scholars consider influential in shaping the perception of gender-based violence (e.g., Davidson & Gervais, 2015; Fernández-Fuertes et al., 2018; Fasanelli et al., 2020; Guerra-Marmolejo et al., 2021).

Concerning the correlational findings, our results generally aligned with the previous psychosocial literature (e.g., Jozkowski et al., 2017; Zaikman & Marks, 2014). However, with specific regard to body surveillance, findings at both Time 0 and Time 1 revealed a positive correlation with the display of positive attitudes toward sexual consent, and a negative correlation with hostile sexism. Concerning the first result, both body surveillance and the propensity to establish explicit sexual consent may be rooted in a heightened sensitivity to the issue of control, referring both to individual bodily appearance and to the preservation of agency during sexual activity. Concerning the second finding, the negative association between body surveillance and hostile sexism may reflect individuals’ perceived necessity to conform to societal standards, in order to gain social approval (Salomon & Brown, 2019). Indeed, while benevolent sexism still appears to be widely accepted (Connelly & Heesacker, 2012), in recent years hostile sexism has been increasingly stigmatized. However, the correlational nature of these results does not allow for any causal inference and thus requires to be further investigated. 

In accordance with our hypotheses, data showed a significant decrease in body surveillance, benevolent sexism, and sexual double standard between T0 and T1 after participating in the intervention at school, thereby confirming hypotheses H1, H2b, and H3 for both adolescent girls and boys, respectively. In particular, the changes observed in body surveillance highlight the importance of fostering a collective reflection on the socially constructed nature of bodily perception, physical sensations, and, above all, the gendered norms perpetuated by media discourses regarding how human bodies should appear (Harper & Tiggemann, 2008; Rollero, 2015; Skowronski et al., 2021). This process may facilitate, in turn, the development of a more authentic connection with one’s bodily experiences, particularly in contexts where the body plays a central role, such as intimate and sexual encounters. In addition, it is noticeable that these variables continue to differ between boys and girls despite their decline, with the latter showing lower values at both T0 and T1. Even though the values for male students remain higher, a change can be associated with participation in the project for both genders.

In contrast, participants’ levels of hostile sexism (H2a) and positive attitudes toward giving consent (H4) did not change significantly following the conclusion of the intervention. Concerning H2, this result may be partly attributed to adolescents’ varying levels of pre-existing awareness regarding the two forms of sexism addressed in the intervention. Indeed, hostile sexism is generally expressed through overtly antagonistic attitudes toward women and, in recent decades, has seen a marked decline in social acceptance, particularly among younger generations (Gomes et al., 2022). Benevolent sexism, by contrast, typically manifests in more subtle forms and is still perceived as more socially legitimate, making it more difficult to recognize and critically evaluate (Dai, 2024). Therefore, it is plausible that the intervention offered participants an opportunity to critically reflect, perhaps for the first time, on some of their implicit benevolent sexist beliefs, thereby fostering a process of deconstruction.

Finally, with regard to H4, it should be noted that at T1, unlike at T0, the relationships between positive attitudes towards consent and the other dimensions analyzed are weaker. This suggests that the intervention may have facilitated a critical reflection that undermines the link between representations of gender relations and the legitimacy of TDV.

### Limitations

This study has some limitations. First, the single-group, test–retest design inherently prevents drawing strong causal inferences. Indeed, without a control or comparison group, the observed changes cannot be certainly attributed to the intervention rather than to other confounding factors. Future studies may employ randomized controlled designs to more rigorously assess causal relationships and isolate the specific effects of the intervention. Additionally, the small sample size limits the statistical power of the comparative analysis between T0 and T1 and restricts the generalizability of the findings. Moreover, although the present study employed primarily univariate analyses to assess pre–post changes in the target variables, we acknowledge the potential value of incorporating multivariate approaches—particularly those that explore mediating and moderating mechanisms—in future research. However, given the relatively limited sample size in our study, we opted not to conduct more complex statistical procedures, as small samples can lead to biased estimates and unstable standard errors in multivariate models (Schönbrodt & Perugini, 2013). We encourage future evaluations of similar interventions, especially those with larger and more diverse samples, to adopt multivariate frameworks that are theoretically grounded and statistically robust.

Regarding the structure of the questionnaire, in order to minimize participant fatigue, we did not include specific measures to assess individual social desirability bias. Future studies should consider incorporating validated measures of social desirability in order to enhance the validity of self-reported data. Moreover, the questionnaire did not include any specific quality control indicators (e.g., attention check items); hence, although data were collected in a supervised classroom setting, we were not able to ensure that participants did not engage in mindless responding. Furthermore, the instruments we used to measure the dimensions involved, which were developed and validated in previous studies, were not specifically designed for this intervention. As a result, some terms and concepts may not fully align with the verbal expressions employed by the team of professionals who led the school intervention. For example, while the notion of consent was addressed broadly throughout the intervention, the questionnaire explored the more narrowly defined concept of sexual consent (Willis et al., 2019). Finally, some of the dimensions addressed by the school intervention may require more time to produce measurable results. Indeed, while short interventions can raise awareness and promote initial reflection on the topics involved, the attitudes and myths surrounding TDV are strongly rooted in individuals’ core beliefs and may not fully shift through only a few sessions. The literature on brief school-based interventions indicates that, although early changes in attitudes can be promising, more intimate or deeply held beliefs typically require longer engagements to change substantially (e.g., Giannakopoulos et al., 2012). Nonetheless, brief programs can initiate cognitive and emotional processes that may continue to unfold and strengthen over time. A follow-up study conducted several months post-intervention could reveal slower and more profound changes in participants’ attitudes and perception of TDV, thereby providing a more comprehensive understanding of the intervention’s sustained impact.

## 5. Conclusions

Despite these limitations, this study offers a valuable contribution by providing a scientific evaluation of the intervention. By addressing the underlying social, emotional, and cognitive factors that contribute to harmful gender norms and relationship dynamics, such interventions can play a pivotal role in promoting safe and respectful adolescent dating relationships. In this regard, although several scholars (e.g., DeGue et al., 2014) have emphasized the importance of implementing primary prevention programs in schools to reduce adolescents’ risk of gender-based violence, rigorous evaluations of such interventions remain limited. Therefore, systematic assessment is essential not only to refine and enhance program effectiveness, but also to guide the strategic allocation of resources, including public and private funding.

## Figures and Tables

**Table 1:** Pearson’s correlations between study variables at Time 0.

	1.	2.	3.	4.	5.
1. Body Surveillance					
2. Benevolent Sexism	−0.00				
3. Hostile Sexism	−0.24 **	0.30 **			
4. Sexual Double Standard	−0.07	0.26 **	0.63 **		
5. Lack of Perceived Ctrl	−0.14	0.36 **	0.37 **	0.30 **	
6. Positive Attit. to Consent	0.20 *	−0.28 **	−0.45 **	−0.39 **	−0.50 **

** *p* < 0.001; * *p* < 0.01.

**Table 2:** Gender differences on the study variables at Time 0: means, standard deviations (SDs), and *t*-test scores.

		Mean	SD	*t*	Cohen’s d
Body Surveillance	Women	5.41	0.93	6.61 **	1.01
	Men	4.43	1.06		
Benevolent Sexism	Women	2.23	1.06	0.42	0.95
	Men	2.17	0.86		
Hostile Sexism	Women	1.38	0.91	−8.22 **	1.01
	Men	2.61	1.08		
Sexual Double Standard	Women	0.81	0.51	−6.73 **	0.50
	Men	1.30	0.49		
Lack of Perceived Ctrl	Women	2.73	1.27	−2.89 *	1.21
	Men	3.25	1.16		
Positive Attit. to Consent	Women	5.32	1.22	4.57 **	1.13
	Men	4.56	1.06		

** *p* < 0.01; * *p* < 0.05.

**Table 3:** Pearson’s correlations between study variables at Time 1.

	1.	2.	3.	4.	5.
1. Body Surveillance					
2. Benevolent Sexism	0.01				
3. Hostile Sexism	−0.21 *	0.40 **			
4. SDS	−0.10	0.45 **	0.61 **		
5. Lack of Perceived Ctrl	−0.11	0.38 **	0.41 **	0.43 **	
6. Positive Attit. to Consent	0.14	−0.18 *	−0.47 **	−0.36 **	−0.48 **

** *p* < 0.001; * *p* < 0.01.

**Table 4:** Gender differences on the study variables at Time 1: means, standard deviations (SDs), and *t*-test scores.

		Mean	SD	*t*	Cohen’s d
Body Surveillance	Women	5.16	0.95	5.20 **	0.96
	Men	4.36	1.00		
Benevolent Sexism	Women	1.83	1.00	−0.69	0.96
	Men	1.94	0.93		
Hostile Sexism	Women	1.19	0.96	−8.14 **	1.01
	Men	2.47	1.04		
Sexual Double Standard	Women	0.69	0.56	−5.57 **	0.54
	Men	1.16	0.52		
Lack of Perceived Ctrl	Women	2.63	1.18	−3.36 **	1.09
	Men	3.20	1.01		
Positive Attit. to Consent	Women	5.29	1.32	3.72 **	1.12
	Men	4.63	0.64		

** *p* < 0.01.

**Table 5:** Differences between Time 0 and Time 1 on the study variables: means, standard deviations (SDs), and *t*-test scores.

		Mean	SD	*t*	Cohen’s d
Body Surveillance	Time 0	4.86	1.11	2.03 *	1.11
	Time 1	4.62	1.18		
Benevolent Sexism	Time 0	2.18	0.95	2.76 **	0.96
	Time 1	1.90	0.96		
Hostile Sexism	Time 0	2.09	1.20	1.25	1.20
	Time 1	1.93	1.19		
Sexual Double Standard	Time 0	1.09	0.57	1.99 *	0.58
	Time 1	0.98	0.59		
Lack of Perceived Ctrl	Time 0	2.99	1.18	0.30	1.19
	Time 1	2.95	1.01		
Positive Attit. to Consent	Time 0	4.92	1.21	0.09	1.19
	Time 1	4.91	1.17		

** *p* < 0.01; * *p* < 0.05.

## Data Availability

Research data are available upon request to the corresponding author.
